# Transactions of the Royal Academy of Medicine in Ireland

**Published:** 1892-06

**Authors:** 


*Transactions of the Royal Academy of Medicine in Ireland.* Vol. VIII., pp. xl., 535. Vol. IX., pp. xxxix., 516. Dublin: FANNIN & Co. 1890—1891.

These volumes are full of articles on the leading topics of our time, are well worthy of the school from which they emanate, and will well repay the reader for the time expended in their perusal. Each represents the most valuable portion of a year's work done by the fellows and members of the Royal Medical Society of Dublin, and all the world knows how instructive and entertaining they are.

